# Association of Mediterranean Diet Adherence with Disease Progression Characteristics, Lifestyle Factors and Overall Survival in Gastric Cancer Patients

**DOI:** 10.3390/medsci11040074

**Published:** 2023-11-15

**Authors:** Eleni Pavlidou, Sousana K. Papadopoulou, Maria Tolia, Maria Mentzelou, Nikolaos Tsoukalas, Olga Alexatou, Theodora Tsiouda, Gerasimos Tsourouflis, Evmorfia Psara, Vasileios Bikos, Nikolaos Kavantzas, Ioly Kotta-Loizou, Antonios Dakanalis, Theofanis Vorvolakos, Constantinos Giaginis

**Affiliations:** 1 Department of Food Science and Nutrition, School of the Environment, University of the Aegean, 81400 Lemnos, Greece; elen.p.pavl@gmail.com (E.P.); maria.mentzelou@hotmail.com (M.M.); rd.olga.alexatou@gmail.com (O.A.); fnsd21013@fns.aegean.gr (E.P.); cgiaginis@aegean.gr (C.G.); 2 Department of Nutritional Sciences and Dietetics, Faculty of Health Sciences, International Hellenic University, 57400 Thessaloniki, Greece; 3 Department of Radiotherapy, Faculty of Medicine, School of Health Sciences, University of Crete, 71003 Heraklion, Crete, Greece; mariatolia1@gmail.com; 4 Department of Oncology, 401 General Army Hospital of Athens, 401 Geniko Stratiotiko Nosokomeio, 11525 Athens, Greece; tsoukn@yahoo.gr; 5 Oncology Department, “Theageneio” Anticancer Hospital, 54639 Thessaloniki, Greece; doratsiouda@yahoo.gr (T.T.); billbik@yahoo.com (V.B.); 6 Second Department of Propedeutic Surgery, School of Medicine, National and Kapodistrian University of Athens, 11527 Athens, Greece; gtsourouflis@med.uoa.gr; 7 First Department of Pathology, School of Medicine, National and Kapodistrian University of Athens, Athens General Hospital “Laikon”, 11527 Athens, Greece; nkavantz@med.uoa.gr; 8 Department of Life Sciences, Faculty of Natural Sciences, Imperial College London, South Kensington Campus, London SW7 2AZ, UK; i.kotta-loizou13@imperial.ac.uk; 9 Department of Mental Health, Fondazione IRCCS San Gerardo dei Tintori, 20900 Monza, Italy; antonios.dakanalis@unimib.it; 10 School of Medicine and Surgery, University of Milano-Bicocca, 20900 Monza, Italy; 11 Department of Psychiatry, School of Medicine, Democritus University of Thrace, 68100 Alexandroupolis, Greece; tvorvola@med.duth.gr

**Keywords:** gastric cancer, Mediterranean diet, quality of life, physical activity, sleep quality, depression, anxiety, body mass index, survival, disease progression

## Abstract

Background: The Mediterranean diet (MD) exerts a protective effect against cancer development and progression; however, the evaluation of its impact on gastric cancer still remains quite scarce. The present study aims to evaluate the association of MD adherence during the lifespan with disease progression characteristics, lifestyle factors and overall survival in gastric carcinoma patients. Methods: This is an observational, cross-sectional study conducted on 186 gastric cancer patients followed up for a median time interval of 57 months or until death due to cancer disease. Tumor histopathological characteristics were retrieved from patients’ medical records, while validated questionnaires assessing, immediately after the time of diagnosis, health-related quality of life, physical activity levels, sleep quality, depression, anxiety and MD adherence during the lifespan were used. Results: Higher MD adherence during the lifespan was significantly associated with younger patients (*p* = 0.0106), regular smoking (*p* < 0.0001), abnormal BMI status (*p* < 0.0001), intestinal-type gastric carcinoma (*p* = 0.0111), high tumor histopathological grade (*p* < 0.0001) and earlier disease stage (*p* < 0.0001). Moreover, patients with elevated MD adherence during their lifespan showed significantly better health-related quality of life (*p* < 0.0001), higher physical activity levels (*p* < 0.0001), more adequate sleep quality (*p* < 0.0001) and lower prevalence of depression (*p* = 0.0003) and anxiety (*p* = 0.0006) compared to those with reduced MD adherence. In multiple regression analysis, elevated MD compliance during the lifespan was independently correlated with longer overall patient survival after adjustment for several confounders (Cox regression analysis, *p* = 0.0001). Conclusions: Higher MD adherence during the lifespan was associated with less advanced tumor histopathology characteristics and favorable mental and physical lifestyle factors. Moreover, higher MD adherence during the lifespan was also independently correlated with longer overall survival in gastric carcinoma patients. Thus, adopting a healthy dietary pattern like the MD during the lifespan may act as a preventive agent in combination with a healthy lifestyle against gastric cancer development and progression.

## 1. Introduction

Based on Global Cancer Inventory (GLOBOCAN) estimations in 2018, the new cancer cases reached 18.1 million, leading to the deaths of 9.6 million persons [1,2,3]. Moreover, cancer constitutes the second-most usual cause of premature death after cardiovascular diseases [1,2,3]. It is already known that about 5–10% of all cancer cases have been ascribed to genetic factors, while the etiopathogenesis of the resting 90–95% have been attributed to deleterious exogenous environmental factors or an unhealthy diet and lifestyle during the lifespan [2,3]. An unhealthy lifestyle can include an unbalanced diet, excessive body weight, physical inactivity, overconsumption of alcohol and tobacco, chemical exposure and obesity [4,5]. Many types of cancers can have a more favorable prognosis and a lower aggressive progression if the modifiable risk factors above can be improved [6].

Gastric cancer constitutes a severe health issue as it ranks first in gastrointestinal cancers, sixth in incidence and second in cancer deaths [7]. Based on GLOBOCAN records in 2018, 1,033,700 novel cases and 782,700 deaths were globally noted due to gastric cancer [7]. As far as their anatomic location is concerned, gastric cancers (GCs) are classified as gastric cardia adenocarcinomas (GCAs) and gastric non-cardia adenocarcinomas (GNCAs) [8]. As far as their histological classification is concerned, two types can be diagnosed, intestinal and diffuse. Intestinal carcinomas are distinguished by visible glands and cohesion among cancer cells [8,9]. The diffuse histological type comprises weakly cohesive cells, diffusely infiltrating the gastric wall with small or no gland formation [8,9].

The prevalence of gastric non-cardia carcinoma is gradually decreasing worldwide, which may be ascribed to the lower incidence of *Helicobacter pylori* infection, a well-recognized risk factor for GNCAs [10,11]. In contrast, gastric cardia carcinoma frequency is constantly growing, which is in accordance with the rise in obesity and adherence to an unhealthy Western lifestyle worldwide [10,11]. Based on the World Cancer Research Fund (WCRF), nutritional status seems to be very important for the prognosis of cancer risk [12]. Moreover, nutritional status has been shown to influence post-treatment disease progression and patients’ survival [12]. An elevated intake of fruits, vegetables and whole grains and a low consumption of red and processed meats have been shown to decrease cancer risk [13]. The role of nutrition is also very crucial as a leading contributor to cancer and an efficient strategy in secondary/tertiary prevention or as a complementary tool of an adjunctive treatment [2,14]. Additionally, malnutrition is worsened in cancer disease due to elevated catabolism, which is ascribed to immune response dysregulation and metabolic impairments, leading to anorexia, raised energy expenditure and body weight decline [15].

Due to the additive or synergistic impact of foods, nutritional models have been implemented as a tool in the field of nutritional epidemiology [16,17]. A Western diet includes the high consumption of refined grains, processed and red meats, desserts, saturated fats and sweets, and it is associated with an elevated glycemic load that can result in an increase in body weight fat mass [18]. A recent systematic review that included 29 prospective studies found a strong relationship between unfavorable dietary patterns like the Western diet and cancer development [18]. Processed foods, red meat products, alcohol, foods containing high dietary fat and dietary cholesterol can also increase the probability of gastric carcinogenesis [19]. In contrast, the elevated consumption of fruits, vegetables, whole grains, nuts and a low-salt diet might exert a preventive impact against gastric cancer [19].

The dietary pattern of the Mediterranean diet (MD) was firstly explored by Keys and co-workers, who observed a considerably smaller incidence of coronary heart disease in countries bordering the Mediterranean basin (Cyprus, Greece, France and Italy) [2,20]. The existing international bibliographical evidence has supported that the MD may represent a dietary pattern suitable to preventing and managing non-communicable disorders [2,20]. The MD mainly constitutes a plant-based dietary pattern, which includes an elevated intake of fruits, vegetables, nuts, legumes, fish, cereals containing whole grains and extra virgin olive oil, a modest intake of red wine, reduced consumption of red and processed meats, and moderate consumption of eggs as well as dairy products [21]. Many systematic reviews and meta-analyses of observational surveys have also reinforced that the MD may exert protective effects against cancer morbidity and mortality [2,20,22]. Greater MD compliance during the lifespan has been linked with lower probability of GCA, GNCA and total GC; however, many of the currently available data are non-significant or conflicting, especially those concerning the distinct gastric carcinoma subtypes [8]. Moreover, several lifestyle factors such as physical activity, health-related quality of life, depression, as well as anxiety beyond or in combination with nutritional habits may influence the outcome of cancer patients; however, the available data regarding gastric cancer remain scarce and inconclusive [5,9,11,14].

Considering the previously available evidence, the present observational, cross-sectional study aims to evaluate the MD compliance in gastric carcinoma patients during their lifespan, while also evaluating its association with disease progression characteristics, lifestyle factors as well as its effect on patients’ prognosis.

## 2. Methods

### 2.1. Study Design

One hundred and eighty-six (186) consecutive gastric carcinoma patients were enrolled in the current survey immediately after cancer diagnosis. Enrollment in the survey started at the time at which biopsy results were announced to the patients between the interval of October 2011 and November 2018. The enrolled patients had not followed any anticancer therapy before diagnosis. Patients with a previous history of cancer were not included in the survey. All patients’ data were confidential, and all patients were informed concerning the aim of the survey by signing a consent form. The survey was authorized by the Bioethics Agency of the University of Aegean (protocol code 7/11.9.2011) and was in accordance with the Declaration of Helsinki of the World Medical Association (52nd WMA General Assembly, Edinburgh, UK).

Concerning histopathologic analysis and TNM stage data, those were retrieved by the participants’ medical records in which distant metastases were established by computed tomography imaging. The participants were followed up for a median period of 57 months or until death due to gastric cancer. Overall survival was outlined at the time interval between the diagnosis date and the death date due to gastric cancer (complete event) or the last date of contact that the participant was known to be alive and disease-free (censored). The participants were followed up about every six months by hospital visits and examinations.

### 2.2. Questionnaire and Lifestyle Factor Evaluations

The Short Form Healthy Survey (SF-36) questionnaire was utilized to assess Health-Related Quality of Life (HRQOL) immediately after the time of diagnosis. This questionnaire includes 36 items, assessing the health state on 8 subscales [23]. The initial four subscales estimate physical HRQOL: physical functioning, physical role limitations, bodily pain and general health perception. Then, four subscales estimate mental HRQOL: role limitations because of emotional troubles, vitality, mental health and social functioning. Concerning the above subscales, a score ranging from 0 (worst) to 100 (best) is determined [23].

The International Physical Activity Questionnaire (IPAQ) was used to evaluate physical activity levels immediately after the time of diagnosis. In this questionnaire, participants report their frequency of exercise in a usual week. This self-administered questionnaire, utilized globally, determines their whole physical activity during the previous week, asking participants to classify it as low, modest or high [24]. The IPAQ tool has been used for evaluations in both developed and developing countries, representing favorable consistency and sufficient validity properties, which is equal to other self-reported PAQs [24].

The Pittsburgh Sleep Quality Index (PSQI) was used to evaluate sleep quality immediately after the time of diagnosis. This questionnaire contains nineteen items scored on a 4-point scale (0–3) and classified into seven components (sleep quality, inactivity, sleep duration, usual sleep effectiveness, sleep impairment, treatment with sleeping medication and daytime disfunction) [25]. The items’ points in each component were added and transformed into component scores rating from 0 (better) to 3 (worse) based on the relative recommendations [25]. Total PSQI scores were calculated as the summation of 7 component scores rating from 0 to 21, in which a greater score showed a worse condition. An overall PSQI score of <5 is indicatory of adequate sleep quality [25].

The Beck Depression Inventory (BDI-II) was used to assess the depression of the patients immediately after the time of diagnosis. This questionnaire includes twenty-one components of statements and constitutes one of the most widely used psychometric tests for determining the severity of depression symptomatology [26]. BDI-II includes items associated with depressive symptoms such as hopelessness and irritability, cognitions like guilt or emotions of being punished, and physical symptoms such as fatigue, weight decline and lack of interest in sex [26]. The BDI-II has been considered as a greatly suitable psychometric tool, presenting sufficient reliability and capability to differentiate depressed from non-depressed people, while indicating enhanced concurrent, content and structural power. Based on the currently existing psychometric evidence, the BDI-II has been recognized as a cost-effective questionnaire for evaluating the severity of depression symptomatology, with broad applicability in research and clinical practice worldwide [26].

The six-item short-form State-Trait Anxiety Inventory (STAI-6) was applied for assessing the anxiety of patients immediately after the time of diagnosis [27]. This is a reliable and valid tool with adequate consistency and validity and with accuracy in response to variations in state anxiety. It is also likely to increase answer rates and decrease the number of response errors and unanswered items, thus improving the validity and generalizability of any results [27].

MD compliance during the lifespan was determined immediately after the time of diagnosis based on the Mediterranean Diet Score (MedDietScore) [28]. The questionnaire effectively assesses the frequency consumptions of 11 selected food categories based on a MedDietScore index [28]. In every question, there are six possible responses, ranging from 0 to 5, related with the level of adherence to every foodstuff category. The sum of the eleven questions leads to a score from 0 to 55; a greater score signifies higher adherence to the MD. For cereals, potatoes, fruits, vegetables, dairy and olive oil, the levels of six possible responses are adapted based on daily consumption [28]. For legumes, seafood, red meat and poultry, the levels of six probable answers are adapted based on weekly consumption [28].

Qualified personnel, nutritionists and dietitians systematically explained to the gastric carcinoma patients the components of the questionnaires to ensure consistent answers [29]. A one-on-one interview was utilized during which every interviewer promptly connected with the participants based on the assembled questionnaires to reduce recall bias. Trained nutritionists and dietitians measured anthropometry parameters such as body mass index (BMI) immediately after the time of diagnosis [29]. Body weight was measured utilizing the same electronic scale, and height was measured by a portable stadiometer (Charder HM200P; Medi-Shop, Greece). It should be emphasized that all lifestyle factors such as quality of life, physical activity, sleep quality, depression, anxiety and MD adherence were assessed immediately after the time of diagnosis, while no medical recommendations were provided for all of the above lifestyle factors during their follow-up.

### 2.3. Statistical Analysis

Student’s *t*-test and one-way ANOVA were used to analyze the continuous variables followed by their normal distribution, which was tested by utilizing the Kolmogorov–Smirnov test. Chi-squared testing was applied for categorical variables. The quantitative normally distributed continuous variables were represented as the mean value ± standard deviation (SD), and the qualitative variables were represented as absolute or relative frequencies. The quantitative non-normally distributed continuous variables were represented as median values (interquartile range, IQR). Survival curves were created by applying the Kaplan–Meier method and the differences between the curves were compared by the log-rank test. Cox proportional hazards regression analysis was applied for assessing the relation of MD compliance during the lifespan with overall patients’ survival, at the multivariate level, including several parameters as potential confounding factors. A *p*-value < 0.05 was used as the limit of statistical significance at a confidence interval (CI) equal to 95%. Statistica 10.0 software was applied for all statistical analyses (Informer Technologies, Inc., Hamburg, Germany).

## 3. Results

### 3.1. Descriptive Statistics of the Study Population

Table 1 includes the descriptive statistics of the study population. In total, 186 patients aged 39 to 88 years old (mean ± SD: 67.6 ± 8.9 years) were assigned to the study. Concerning gender, 66.7% of the enrolled patients were men and 33.3% were women. A total of 60.2% of the gastric patients were never smokers and 39.8% of them were regular smokers. The mean BMI of the participants was 24.1 ± 5.4 kg/m^2^. According to the BMI classification, 25.3% of the patients were underweight, 33.9% had a normal weight, 24.7% were overweight and 16.1% were affected by obesity (Table 1).

As far as tumor histopathological type was concerned, 47.3% of patients were diagnosed with intestinal gastric carcinoma and 52.70% had diffuse gastric carcinoma (Table 1). Regarding the grade of tumor differentiation, 5.4% of patients had a low tumor grade, 45.1%, moderate, and 49.5% presented a high tumor grade (Table 1).

Based on TNM staging, 29.0% of patients were diagnosed with stage I gastric cancer, 21.5% of them had stage II disease, 35.5% exhibited stage III and the remaining 14.0% of the enrolled patients had stage IV gastric cancer (Table 1). Concerning tumor size (pT), 11.8% of participants were categorized as pT1, 32.3% of patients, as pT2, 45.2% of patients, as pT3, and 10.7% of patients, as pT4 (Table 1). Concerning the presence of lymph node metastasis, 35.5% of patients were classified as pN0, 59.1% of patients, as pN1, and 5.4% of patients, as pN2. Regarding organ metastasis, 88.2% of patients had no metastatic disease (pM0), whereas 11.8% had at least one organ metastasis (pM1) (Table 1).

Physical activity levels were directly categorized based on the IPAQ. In fact, 38.2% of participants exhibited low physical activity levels, 33.3% of them showed moderate physical activity levels and 28.5% had enhanced physical activity levels (Table 1). The mean health-related quality of life (EORTC QLQ-C30) score was 53/100 (IQR: 42/100–91/100), rated from 31 to 86. In fact, 50.5% of patients exhibited a low health-related quality of life (HRQOL, score < 53/100) and 49.5% exhibited an enhanced quality of life (HRQOL, score ≥ 53/100) next to dichotomization based on the mean value (Table 1). Concerning sleep quality assessed by the PSQI questionnaire, 40.3% of patients exhibited inadequate sleep quality and 59.7% showed adequate sleep quality (Table 1). A total of 43.0% of the gastric patients were diagnosed with depression and 32.3% of them showed anxiety symptomatology (Table 1).

Regarding MD adherence during the lifespan, assessed by the MedDietScore, the median score was 26 (IQR: 24–29) and ranged from 18 to 35. The level of compliance to the MD was classified into quartiles as very low MD adherence (25.8%), low MD adherence (25.3%), moderate MD adherence (24.7%) and high MD adherence (24.2%) (Table 1).

After the final follow-up, 98 patients (52.7%) were dead due to gastric cancer and 88 patients (47.3%) were alive. The median overall survival time of the patients was 57 months (IQR: 16–90 months) and ranged between 3 and 125 months (Table 1).

### 3.2. Associations of Mediterranean Diet (MD) Compliance with Disease Progression and Lifestyle Factors of Gastric Patients’ Characteristics

Younger patients showed significantly higher MD adherence during their lifespan than older gastric carcinoma patients (Table 2, *p* = 0.0106). Male patients showed a higher compliance to the MD during their lifespan compared to female patients, but at a non-significant level (Table 2, *p* = 0.1378). Never smoker gastric patients were significantly associated with greater MD compliance (Table 2, *p* < 0.0001). BMI as a continuous variable was significantly gradually decreased as the adherence to MD increased (Table 2, *p* = 0.0023). According to BMI status, underweight and obese patients had significantly lower compliance to the MD during their lifespan compared to normal-weight and overweight patients (Table 2, *p* < 0.0001).

Individuals with intestinal gastric carcinoma showed significantly higher MD adherence during their lifespan compared to those with diffuse gastric carcinoma (Table 2, *p* = 0.0111). MD adherence was considerably positively increased with tumor grade of differentiation (Table 2, *p* < 0.0001). Lower MD adherence during the lifespan was considerably related with a more progressive histopathological stage, higher tumor size and lymph node metastasis positivity (Table 2, *p* < 0.0001, *p* = 0.0003 and *p* = 0.0201, respectively). Lower MD adherence during the lifespan was more often noted in participants presenting distance organ metastasis (pM1), but at a non-significant level (Table 2, *p* = 0.1964).

Patients presenting elevated MD compliance during their lifespan exhibited considerably higher physical activity levels compared to those presenting lower MD compliance (Table 2, *p* < 0.0001). Moreover, patients presenting elevated MD adherence showed considerably favorable health-related quality of life compared to those with decreased MD compliance (Table 2, *p* < 0.0001). Patients presenting inadequate sleep quality showed significantly lower MD adherence during their lifespan compared to those having adequate sleep quality (Table 2, *p* < 0.0001). Gastric cancer patients diagnosed with depression significantly showed decreased MD compliance (Table 2, *p* = 0.0003). Accordingly, gastric cancer patients presenting anxiety symptomatology had considerably lower MD compliance (Table 2, *p* = 0.0006). Overall survival times were significantly gradually increased as the adherence to MD increased (Table 2, *p* < 0.0001).

### 3.3. Kaplan–Meier Analysis for Patients’ Overall Survival

**Figure 1:**
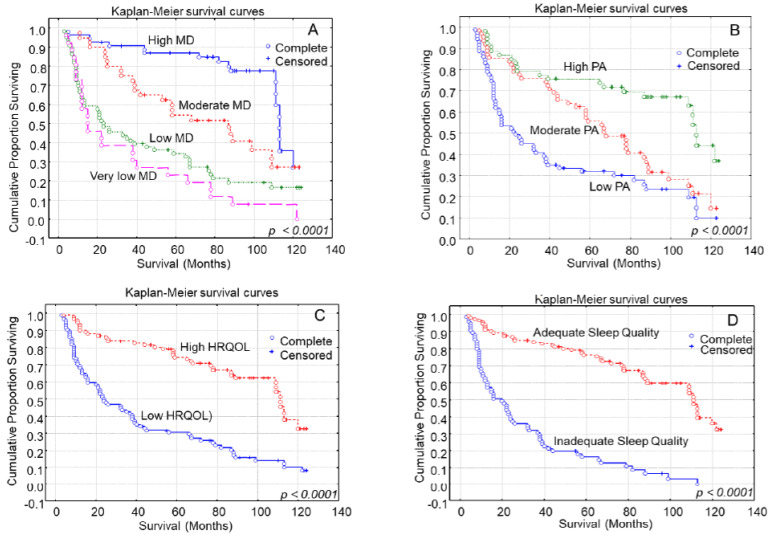
Kaplan–Meier survival analysis based on (**A**) Mediterranean diet, (**B**) physical activity (PA), (**C**) Health-Related Quality of Life (HRQOL) and (**D**) sleep quality in 186 gastric cancer patients for overall survival.

Kaplan–Meier survival curves demonstrated that participants with modest or elevated MD compliance during their lifespan exhibited considerably favorable overall survival times compared to those presenting decreased MD compliance (Figure 1A, Log-rank test, *p* < 0.0001). Younger patients also had significantly longer overall survival times than older patients (Log-rank test, *p* = 0.0009). Underweight and obese patients showed shorter overall survival times compared to normal-weight and overweight patients according to their BMI (Log-rank test, *p* < 0.0001). Never smoker participants had significantly longer survival times compared to regular smoker participants (Log-rank test, *p* = 0.0005). Participants’ gender was not related with overall survival times (Log-rank test, *p* = 0.3842).

Patients with an advanced disease stage had shorter overall survival compared to those with an earlier disease stage (Log-rank test, *p* < 0.0001). Shorter overall survival times were found in participants with a higher tumor size (Log-rank test, *p* = 0.0002), presence of local lymph node involvement (Log-rank test, *p* < 0.0001) and distant organ metastasis (Log-rank test, *p* = 0.0013). Patients presenting intestinal gastric carcinoma showed longer overall survival compared to those with diffuse gastric carcinoma (Log-rank test, *p* = 0.0014). Participants presenting low or moderate tumor grade of differentiation exhibited worse overall survival times than those with a high tumor histopathological grade (Log-rank test, *p* < 0.0001).

Greater physical activity levels were substantially related with favorable overall patient survival times (Figure 1B, Log-rank test, *p* < 0.0001). Patients with better health-related quality of life had higher overall survival times compared to those with decreased health-related quality of life (Figure 1C, Log-rank test, *p* < 0.0001). Patients presenting adequate sleep quality showed longer overall survival times than those presenting inadequate sleep quality (Figure 1D, Log-rank test, *p* < 0.0001).

**Figure 2:**
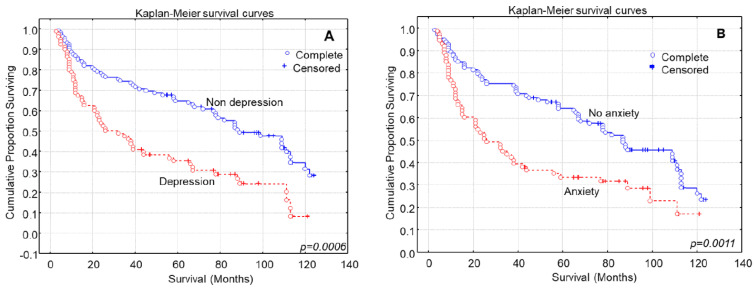
Kaplan–Meier survival analysis based on (**A**) depression and (**B**) anxiety in 186 gastric cancer patients for overall survival.

Gastric cancer patients diagnosed with depression exhibited significantly shorter survival times compared to non-depressed patients (Figure 2A, Log-rank test, *p* = 0.0006). Gastric cancer patients presenting anxiety symptomatology also had shorter overall survival times compared to patients with absence of anxiety symptoms (Figure 2B, Log-rank test, *p* = 0.0011).

### 3.4. Multivariate Analysis of MD Adherence with Patients’ Overall Survival

In the multivariate analysis, MD adherence during the lifespan was independently associated with patients’ overall survival after adjustment for several possible confounders (Table 3, Cox regression analysis, *p* = 0.0001). Patients with elevated MD adherence during their lifespan had a two-fold longer prevalence of longer overall survival times compared to those presenting reduced MD compliance (Table 3, Cox regression analysis, *p* = 0.0001). Normal-weight and overweight patients showed a 35% higher probability of exhibiting high MD adherence compared to underweight and obese patients (Table 3, Cox regression analysis, *p* = 0.0015). Regular smoker patients had a 53% higher likelihood of shorter overall survival times compared to never smoker patients (Table 3, *p* = 0.0036).

Patients with a high tumor grade of differentiation had an 82% higher likelihood of high MD adherence during their lifespan compared to those with a moderate or low tumor histopathological grade of differentiation (Table 3, Cox regression analysis, *p* = 0.0127). Moreover, patients with a low tumor size showed a 43% higher probability of high MD adherence during their lifespan compared to those with a larger tumor size (Table 3, Cox regression analysis, *p* = 0.0029). Patients with elevated physical activity levels had a more than two-fold longer survival time than those presenting reduced physical activity (Table 3, Cox regression analysis, *p* = 0.0008). Patients characterized by better health-related quality of life showed a more than two-fold longer survival time compared to those presenting worse quality of life (Table 3, Cox regression analysis, *p* = 0.0023). A two-fold longer survival time was observed in patients with adequate sleep quality than those presenting inadequate sleep quality (Table 3, Cox regression analysis, *p* = 0.0012). Gastric cancer patients presenting depression and anxiety symptoms had a more than two-fold shorter overall survival compared to those without depression and anxiety symptoms (Table 3, Cox regression analysis, *p* = 0.0025 and *p* = 0.0046, respectively).

Patients’ age, gender, histopathological type, and positive lymph node metastases and distant organ metastases were not independently associated with their overall survival (Table 3, Cox regression analysis, *p* > 0.05). When we used the histopathological stage of pTNM as a variable instead of its components, e.g., tumor size, lymph node metastasis and distant organ metastasis, almost identical results were derived.

## 4. Discussion

This is an observational, cross-sectional study evaluating the disease progression and overall survival in gastric cancer patients according to their adherence to the MD during their lifespan. Worldwide, gastric cancer constitutes the fifth-most frequently occurring cancer and the third leading cause of cancer-associated deaths [21,30,31]. In our study, it was found that participants presenting high MD compliance during their lifespan exhibited a two-fold higher overall survival time compared to those with very low MD adherence. The above results are in accordance with previous surveys supporting that diet-related exposure including inverse relations with gastric cancer risk has been noted for non-starchy vegetables and fruits, extra virgin olive oil and legumes [32,33]. An elevated probability of gastric cancer was also shown for salty and smoked foods, processed meat, and grilled or barbecued meat [32,33]. Thus, it was not surprising that the World Cancer Research Fund (WCRF) Continuous Update Program (CUP) in its 2018 report have documented a 15% higher likelihood of gastric cancer in an analysis comparing the greatest vs. lowest categories of salted fish intake, but with inadequate evidence for a dose–response effect, and a 9% increased risk per 20 g of pickled vegetable intake [10,33].

Moreover, in agreement with our results, a recent systematic review and meta-analysis on the impact of MD compliance in the likelihood of different cancers demonstrated that elevated MD compliance was associated with a lower probability of cancer mortality in the general population and all-cause mortality among cancer survivors as well as with the likelihood of colorectal, head and neck, lung, gastric, liver and bladder cancers [1]. Accordingly, in a longitudinal survey on Dutch gastric cancer patients aged 55–69 years, a healthy lifestyle score (HLS) that was characterized by not smoking, presenting a normal BMI, having high physical activity levels, adopting an MD pattern and having no or decreased alcohol consumption indicated a substantial inverse association with the probability of gastric cancer, in a linear fashion [34]. Another study further evaluated whether nutritional support could improve the prognosis of individuals presenting advanced gastric cancer [35]. More to the point, patients with a Nutritional Risk Screening (NRS) ≥ 3 showed a considerably greater proportion of stage IV diseases, increased concentrations of inflammatory C-reactive protein (CRP) and hypoproteinemia [35]. Median survival was considerably reduced in NRS < 3 patients, while multivariate analysis confirmed that NRS status was independently associated with the prognosis of gastric cancer patients [35]. Especially for stage IV patients with an NRS ≥ 3, nutritional support could contribute to the improvement in patients’ prognosis [35]. As expected from previous research, a meta-analysis supported evidence that elevated MD compliance was related with a considerable decrease concerning the probability of overall cancer mortality (by approximately 13%) and a reduced prevalence of gastric cancer (by 27%) [2]. However, a systematic literature search on 29 prospective studies revealed that there is currently inadequate evidence supporting a relation between post-diagnostic food choices/dietary patterns and survival outcomes in individuals with gastric cancer [18]. Thus, additional research is strongly recommended to investigate the impact of post-diagnostic nutritional habits in gastric cancer [18].

An updated meta-analysis on the relation between MD and cancer prevalence and mortality retrieved data from 29 observational studies and randomized clinical trials [36]. This meta-analysis revealed an association between higher MD compliance and a lower probability of overall mortality, cardiovascular disorders, coronary heart disease, myocardial infarction, overall cancer prevalence, neurodegenerative disorders and diabetes mellitus [36]. Moreover, higher MD compliance was related not only with an elevated time to relapse, but also with a greater nourishment status [36]. Although the majority of the currently available surveys have focused on the relation between MD adherence and a lower probability of cancer, only a few studies, such as the current one, have investigated its association with patients’ survival or relapse.

Currently, adherence to the MD during the lifespan in Greece has gradually decreased [37]. A recent meta-analysis, including surveys published during the decade of 2011–2021, indicated that a high-quality diet (via the Healthy Eating Index, HEI-2005 and HEI-2015, and Alternative Healthy Eating Index, AHEI) after diagnosis was related with a 23% decreased all-cause mortality, but not cancer-specific mortality [38]. In addition, improving nutritional habits by 10 points may reduce all-cause mortality by 9% [38]. Foodstuffs that are included in the MD constitute a conglomeration of several bioactive ingredients, which are characterized by strong anticancer, anti-inflammatory and antioxidant properties, and their molecular mechanisms especially of anticancer action have been documented in preliminary clinical surveys [39].

Health-related quality of life can be considered as patients’ subjective view of the effects of their disorders and their therapies in daily life, including their physical, psychological and social functioning and well-being [40]. Our findings showed a strong connection between higher physical activity and better health-related quality of life with longer overall patient survival times, which was in accordance with a comprehensive search performed in Medline, exploring 35 prospective cohorts and 14 randomized controlled trials [38]. The above studies supported evidence that an intervention that combines nutritional and physical activity advice and support may improve overall quality of life among cancer survivors; however, the available data have remained inadequate so far to derive accurate conclusions or propose recommendations [38].

Quality of life is an exceptionally complicated concept and includes diverse psychological, physical, social and cultural aspects of well-being and health-related quality of life. Healthy nutritional habits exert a crucial impact on several aspects of mental and physical health as well as in the prevention and treatment of non-communicable disorders [41]. Concerning gastric cancer, poor nutritional status has independently been associated with the prognosis of relapse and disease-free survival and shorter overall survival [42,43,44]. The MD seems helpful in the prevention of chronic diseases, like cancer. In accordance with our bibliographical results, greater adherence to the MD has been related with a substantial improvement in general psychological and physical health and lower overall mortality [41,45,46].

Sleep habits are crucial in human health. They are also associated with strengthening memory, improving vision, maintaining body temperature, and maintaining and recovering energy and restoration of the brain’s energy metabolism [47,48,49]. The quality of one’s diet has been considered to significantly affect sleep quality which in turn affects mental and physical health [47]. Several studies have also highlighted the favorable effect of MD compliance on sleep quality and duration in both adolescence and adulthood in both cross-sectional and prospective cohorts [48,49].

Furthermore, it has been well established that the diagnosis and treatment of cancer are stressful events, which may induce psychological distress in the majority of cancer patients [50]. Notably, a case cohort of 12,664 participants aged over 40 years showed that those with gastric cancer exhibited a greater probability of a new onset of depressive symptoms compared to healthy individuals. Older gastric cancer patients at the age of 60 years exhibited the greatest likelihood of new onset of depressive symptoms than other age groups and non-cancer individuals [50]. Moreover, gastric cancer patients with a history of depression before cancer diagnosis showed an elevated probability of a new onset of depressive symptomatology compared to gastric cancer individuals without an antecedent diagnosis of depression [50]. A prospective study in China also supports the hypothesis that depression is associated with poor survival among gastric cancer patients [51]. From a molecular point of view, depressive symptom severity was significantly higher in gastric cancer patients having higher plasma levels of catecholamines, elevated metastasis-associated in colon cancer-1 (MACC1) oncogene levels and metastasis [52]. This study reinforces the development of novel more effective treatment strategies for improving the outcome and daily performance status of gastric cancer patients with comorbid depression [52].

In addition, based on Hospital Anxiety and Depression Scale (HADS) scores, it was found that the prevalence and severity of anxiety was highly elevated in 200 gastric cancer individuals undergoing surgical resection compared with healthy controls [53]. Notably, the presence of anxiety/depression as well as the symptom severity of anxiety/depression were all negatively related with disease-free survival as well as overall survival in gastric cancer individuals undergoing surgical operation [53]. The above evidence supports the strong demand for psychological counselling and support prior to surgical operation of individuals with gastric cancer [53]. Interestingly, in a recent case–control study, video-based nursing training was effective in reducing the perioperative anxiety and depression of patients undergoing minimally invasive gastrectomy [54]. In this aspect, multidisciplinary cooperative continuous nursing can exert beneficial impacts in individuals with gastric cancer, ameliorate depression, anxiety, postoperative pain and quality of life, and facilitate the self-care ability and satisfaction of patients [55]. Reminiscence therapy-based care was also found to serve as an efficient interventional approach in relieving anxiety and depressive symptoms and improve quality of life in 48 recurrent gastric cancer patients compared to 48 patients receiving usual care during a 12-week intervention with follow-up for 6 months [56]. In a recent case–control study, treatment with enteral nutritional therapy in individuals with gastric cancer in combination with psychiatric medication was found to efficiently improve patient anxiety and depressive symptoms, increasing therapy compliance, improving the nourishment state and enhancing quality of life [57]. A prospective cohort study investigated the longitudinal alteration and possible probability factors of postoperative anxiety and depression symptoms in individuals with gastric cancer undergoing surgical operation [58]. This study clearly demonstrated that postoperative anxiety and depressive symptoms were progressively worsened, associated with a deleterious prognosis, while the most important risk factors included female, a single/divorced/widowed marital status, diabetes, hyperlipidemia, a high tumor size and an advanced TNM stage [58].

Concerning another retrospective clinical study conducted on 34 terminal gastric cancer patients admitted to a palliative care unit, evidence was presented supporting that patients with anxiety exhibited a higher probability of emergency admission, an elevated Numerical Rating Scale result, occurrence of cachexia and neuropsychiatric symptoms, a longer duration of treatment, increased albumin levels and increased glucose amounts [59]. Individuals with neuropsychiatric symptomatology also exhibited a higher probability of emergency admission, better performance status scale, occurrence of dyselectrolytemia and decreased albumin levels. Notably, patients presenting those symptoms exhibited more than seven times a higher risk of death [59]. Another study, including 80 newly diagnosed gastric cancer patients and 80 healthy controls, assessed their anxiety and depression symptoms utilizing the HADS [60]. The HADS anxiety scores and the proportion of anxiety patients were more increased in recurrent gastric cancer patients than in the newly diagnosed individuals with gastric cancer and in the healthy controls [60]. This study clearly showed a higher incidence of anxiety and depression symptoms and their relevant risk factors as age ≥60 years, diabetes and a smaller time to relapse in recurrent gastric cancer patients [60].

Our study has several strengths. The current work constitutes one of the few observational, cross-sectional surveys that explores the impact of MD adherence during the lifespan on disease progression and overall survival in gastric cancer patients by taking into account several lifestyle factors, such as quality of life, physical activity, sleep quality, and depression and anxiety symptoms’ severity. Moreover, in our analysis, we adjusted for a wide range of disease confounders to explore whether MD adherence during the lifespan could independently be associated with gastric cancer progression. In addition, the sample size permitted the assessment of possible interrelationships of nutritional habits with patients’ sex and age and the exploration of the potential relationship between tumor location and morphology. It is also one of the few surveys to have investigated the relationship between the MD and physical activity, quality of life, sleep quality, and depression and anxiety symptoms’ severity in Greece. Moreover, we utilized authorized questionnaires to reliably evaluate the above findings through face-to-face interviews with qualified personnel and detailed presentations of the given questionnaires to minimize recall bias and to increase the validity of patients’ responses. Moreover, the patients under study were followed up for a median interval of 57 months, which reinforced the relation between MD compliance with overall patients’ survival. Another strength of our study concerns the evaluation of several aspects of the mental health of gastric cancer patients in association with their prognosis.

However, our study also has certain limitations. Firstly, the questionnaires requested detailed information and recall bias could not be excluded. It should be emphasized that MD adherence was assessed immediately after the time of diagnosis and concerned the compliance to this dietary pattern during the lifespan of the enrolled patients, and thus, potential recall bias was unavoidable. Moreover, all data concerning the lifestyle of the enrolled patients were made immediately after the time of diagnosis and may have been influenced by the worse psychological state of the participants. The present findings were derived from a Greek population and thus, they cannot be generalized to other ethnicities, especially beyond European populations. Several other confounding factors related with mental health, e.g., perceived stress, and lifestyle factors, e.g., marital status, educational and financial status, of the enrolled gastric cancer patients may have affected the impact of the MD on their disease progression and prognosis. Moreover, no data concerning recurrence-free patients’ survival, as well as the kinds of medication treatment, were available. Lastly, the study design did not permit the derivation of causality effects.

## 5. Conclusions

The present study highlights that higher levels of MD adherence during the lifespan may be associated with a lower prevalence of advanced disease progression and shorter overall survival in gastric cancer patients. Our findings also support that elevated MD compliance during the lifespan is related with greater quality of life, adequate sleep quality, less depression and anxiety symptoms, and higher physical activity levels in gastric cancer patients. However, the precise effects of the MD on disease progression are still under investigation, and future studies are suggested to explore the impacts of various nutritional interventions to prevent gastric cancer development and progression and to prolong patients’ survival. Our findings have emphasized the importance of performing large, prospective, well-designed, randomized, interventional, clinical trials to generate data indicating improvements in cancer-specific outcomes (recurrence, disease-free survival) as a result of these dietary (and other lifestyle) interventions. Initiatives should also be put forth to contribute to health-promoting results and persuade public health policy makers and responsible bodies to better understand cancer-preventing diets and promote changes in nutritional patterns that could decrease cancer risks and improve cancer outcomes.

## Figures and Tables

**Table 1:** Descriptive statistics of the study population.

Parameter, n = 186	Descriptive Statistics
Mean age (years ± SD *)	67.6 ± 8.9
**Gender (n, %)**	
Male	124 (66.7%)
Female	62 (33.3%)
**Smoking (n, %)**	
Regular smoker	122 (60.2%)
Never smoker	74 (39.8%)
Mean BMI (kg/m^2^, SD *)	24.1 ± 5.4
**BMI status (n, %)**	
Underweight	47 (25.3%)
Normal weight	63 (33.9%)
Overweight	46 (24.7%)
Obese	30 (16.1%)
**Histopathological type (n, %)**	
Intestinal gastric carcinoma	88 (47.3%)
Diffuse gastric carcinoma	99 (52.7%)
**Tumor grade of differentiation (n, %)**	
Low	10 (5.4%)
Moderate	84 (45.1%)
High	92 (49.5%)
**Tumor stage (n, %)**	
Stage I	54 (29.0%)
Stage II	40 (21.5%)
Stage III	66 (35.5%)
Stage IV	26 (14.0%)
**Tumor size (n, %)**	
pT1	22 (11.8%)
pT2	60 (32.3%)
pT3	84 (45.2%)
pT4	20 (10.7%)
**Lymph node metastasis (n, %)**	
pN0	66 (35.5%)
pN1	110 (59.1%)
pN2	10 (5.4%)
**Distant metastasis (n, %)**	
pM0	164 (88.2%)
pM1	22 (11.8%)
**International Physical Activity Questionnaire (IPAQ) level (n, %)**	
Low	71 (38.2%)
Moderate	62 (33.3%)
High	53 (28.5%)
**Health-Related Quality of Life (HRQOL) (n, %)**	
Low	94 (50.5%)
High	92 (49.5%)
**Pittsburgh Sleep Quality Index (PSQI) (n, %)**	
Inadequate	75 (40.3%)
Adequate	111 (59.7%)
**Depression (n, %)**	
No	106 (57.0%)
Yes	80 (43.0%)
**Anxiety (n, %)**	
No	113 (60.7%)
Yes	73 (32.3%)
Median Mediterranean diet score (IQR **)	26 (IQR: 24–29)
**Mediterranean diet quartile (n, %)**	
Very low	48 (25.8%)
Low	47 (25.3%)
Moderate	46 (24.7%)
High	45 (24.2%)
Patients’ overall survival (months, IQR **)	57 (IQR: 16–90)

* Standard deviation (SD), ** Interquartile range (IQR).

**Table 2:** Associations between MD adherence and patients’ characteristics and lifestyle factors.

Characteristic, n = 186	Mediterranean Diet (MD) Adherence				
	Very Low	Low	Moderate	High	*p*-Value
Age (years, mean ± SD *)	71 ± 9.5	67.6 ± 7.9	65.9 ± 10.3	65.5 ± 6.5	*p* = 0.0106
**Gender (n, %)**					*p* = 0.1378
Male	29 (60.4%)	28 (59.6%)	31 (67.4%)	36 (80.0%)	
Female	19 (39.6%)	19 (40.4%)	15 (32.6%)	9 (20.0%)	
**Smoking status (n, %)**					*p* < 0.0001
Never smoker	17 (35.4%)	22 (46.8%)	37 (80.4%)	36 (80.0%)	
Regular smoker	31 (64.6%)	25 (53.2%)	9 (19.6%)	9 (20.0%)	
Mean BMI (kg/m^2^ ± SD *)	26.6 ± 8.6	25.7 ± 7.2	24.9 ± 6.5	24.2 ± 6.8	*p* = 0.0023
**BMI class (n, %)**					*p* < 0.0001
Underweight	26 (54.2%)	14 (29.8%)	3 (6.5%)	4 (8.9%)	
Normal weight	7 (14.6%)	15 (31.9%)	18 (39.1%)	23 (51.1%)	
Overweight	2 (4.2%)	9 (19.2%)	20 (43.5%)	15 (33.3%)	
Obese	13 (27.1%)	9 (19.2%)	5 (10.9%)	3 (6.7%)	
**Histopathological type (n, %)**					*p* = 0.0111
Intestinal gastric carcinoma	15 (31.3%)	20 (42.6%)	24 (52.2%)	29 (64.4%)	
Diffuse gastric carcinoma	33 (68.7%)	27 (57.4%)	22 (47.8%)	16 (35.6%)	
**Tumor differentiation grade (n, %)**					*p* < 0.0001
Low	36 (75.0%)	32 (68.1%)	19 (41.3%)	5 (11.1%)	
Moderate	12 (25.0%)	14 (29.8%)	23 (50.0%)	35 (77.8%)	
High	0 (0.0%)	1 (2.1%)	4 (8.7%)	5 (11.1%)	
**Tumor stage (n, %)**					*p* < 0.0001
Stage I	6 (12.5%)	7 (14.9%)	24 (52.2%)	17 (37.8%)	
Stage II	4 (8.3%)	14 (29.8%)	8 (17.4%)	14 (31.1%)	
Stage III	27 (56.3%)	17 (36.2%)	11 (23.9%)	11 (24.4%)	
Stage IV	11 (22.9%)	9 (19.1%)	3 (6.5%)	3 (6.7%)	
**Tumor size (n, %)**					*p* = 0.0003
pT1	2 (4.2%)	3 (6.4%)	10 (21.7%)	7 (15.6%)	
pT2	8 (16.7%)	14 (29.8%)	20 (43.5%)	18 (40.0%)	
pT3	25 (52.1%)	26 (55.3%)	13 (28.3%)	20 (44.4%)	
pT4	13 (27.1%)	4 (8.5%)	3 (6.5%)	0 (0.0%)	
**Presence of lymph node metastasis (n, %)**					*p* = 0.0201
No	12 (25.0%)	12 (25.5%)	19 (41.3%)	23 (51.1%)	
Yes	36 (75.0%)	35 (74.5%)	27 (58.7%)	22 (48.9%)	
**Presence of distant metastasis (n, %)**					*p* = 0.1964
No	40 (83.3%)	39 (83.0%)	43 (93.5%)	42 (93.3%)	
Yes	8 (16.7%)	8 (17.0%)	3 (6.5%)	3 (6.7%)	
**Physical activity (n, %)**					*p* < 0.0001
Low	28 (58.4%)	21 (44.7%)	15 (32.6%)	7 (15.6%)	
Moderate	10 (20.8%)	22 (46.8%)	13 (28.3%)	17 (37.7%)	
High	10 (20.8%)	4 (8.5%)	18 (39.1%)	21 (46.7%)	
**HRQOL (n, %)**					*p* < 0.0001
Low (Below mean value)	37 (77.1%)	28 (59.6%)	17 (37.0%)	12 (26.7%)	
High (Over mean value)	11 (22.9%)	19 (39.4%)	29 (63.0%)	33 (73.3%)	
**PSQI (n, %)**					*p* < 0.0001
Inadequate	36 (75.0%)	31 (66.0%)	6 (13.0%)	2 (4.4%)	
Adequate	12 (25.0%)	16 (44.0%)	40 (87.0%)	43 (95.6%)	
**Depression (n, %)**					*p* = 0.0003
No	17 (35.4%)	21 (44.7%)	34 (73.9%)	34 (75.6%)	
Yes	31 (64.6%)	26 (55.3%)	12 (26.1%)	11 (24.4%)	
**Anxiety (n, %)**					*p* = 0.0006
No	18 (37.5%)	25 (53.2%)	34 (73.9%)	36 (80%)	
Yes	30 (62.5%)	22 (46.8%)	12 (26.1%)	9 (20.0%)	
Patients’ overall survival (months, median, IQR **)	17 (7–29)	34 (21–52)	79 (61–95)	102 (88–116)	*p* < 0.0001

* Standard deviation (SD), ** Interquartile range (IQR).

**Table 3:** Multivariate analysis evaluating whether MD compliance during the lifespan was independently associated with patients’ overall survival.

Characteristic	Overall Survival	
	HR * (95% CI **)	*p*-Value
**Age** (Below/Over mean value)	1.02 (0.63–1.58)	*p* = 0.0922
**Gender** (Male/Female)	1.15 (0.39–1.92)	*p* = 0.3478
**Smoking habit** (Never smoker/Regular smoker)	1.53 (1.25–1.88)	*p* = 0.0036
**BMI** (Normal weight and overweight/Underweight and obese)	1.35 (1.04–1.62)	*p* = 0.0015
**Histological type** (Intestinal/Diffuse)	1.22 (0.70–1.81)	*p* = 0.2237
**Histological grade** (High/Moderate/Low)	1.82 (1.54–2.13)	*p* = 0.0127
**Tumor size** (pT1 + pT2/pT3 + pT4)	1.43 (1.11–1.79)	*p* = 0.0029
**Lymph node metastasis** (No/Yes)	1.21 (0.71–1.83)	*p* = 0.1822
**Distant metastases** (No/Yes)	1.05 (0.29–1.81)	*p* = 0.4893
**Physical activity** (High/Moderate/Low)	2.24 (1.98–2.49)	*p* = 0.0008
**Health-Related Quality of Life** (Over/Below mean value)	2.41 (2.18–2.64)	*p* = 0.0023
**Pittsburg Sleep Quality of Life** (Adequate/Inadequate)	2.02 (1.79–2.29)	*p* = 0.0012
**Depression** (No/Yes)	2.68 (2.47–2.90)	*p* = 0.0025
**Anxiety (No/Yes)**	2.54 (2.29–2.81)	*p* = 0.0046
**MD adherence**		*p* = 0.0001
**Very low (Reference)**	1.00	
**Low**	1.27 (0.97–1.55)	
**Moderate**	1.82 (1.58–2.19)	
**High**	2.01 (1.81–2.23)	

* Hazard ratio: OR, ** CI: Confidence interval.

## Data Availability

The data of this study are available upon request to the corresponding author.

## References

[1] Morze J., Danielewicz A., Przybyłowicz K., Zeng H., Hoffmann G., Schwingshackl L. (2021). An updated systematic review and meta-analysis on adherence to mediterranean diet and risk of cancer. Eur. J. Nutr..

[2] Schwingshackl L., Schwedhelm C., Galbete C., Hoffmann G. (2017). Adherence to Mediterranean Diet and Risk of Cancer: An Updated Systematic Review and Meta-Analysis. Nutrients.

[3] Álvarez-Álvarez L., Vitelli-Storelli F., Rubín-García M., Aragonés N., Ardanaz E., Castaño-Vinyals G., Obón-Santacana M., Dierssen-Sotos T., Salas-Trejo D., Tardón A. (2021). Relationship between the Risk of Gastric Cancer and Adherence to the Mediterranean Diet According to Different Estimators. MCC—Spain Study. Cancers.

[4] Barekzai A.M., Aminianfar A., Mousavi S.M., Esmaillzadeh A. (2022). The Association between Dietary Inflammatory Potential and Gastric Cancer: A Case Control Study. Nutr. Cancer.

[5] Poorolajal J., Moradi L., Mohammadi Y., Cheraghi Z., Gohari-Ensaf F. (2020). Risk factors for stomach cancer: A systematic review and meta-analysis. Epidemiol. Health.

[6] Ligibel J.A., Alfano C.M., Courneya K.S., Demark-Wahnefried W., Burger R.A., Chlebowski R.T., Fabian C.J., Gucalp A., Hershman D.L., Hudson M.M. (2014). American Society of Clinical Oncology position statement on obesity and cancer. J. Clin. Oncol..

[7] Dominguez L.J., Di Bella G., Veronese N., Barbagallo M. (2021). Impact of Mediterranean Diet on Chronic Non-Communicable Diseases and Longevity. Nutrients.

[8] Hu B., El Hajj N., Sittler S., Lammert N., Barnes R., Meloni-Ehrig A. (2012). Gastric cancer: Classification, histology and application of molecular pathology. J. Gastrointest Oncol..

[9] Machlowska J., Baj J., Sitarz M., Maciejewski R., Sitarz R. (2020). Gastric Cancer: Epidemiology, Risk Factors, Classification, Genomic Characteristics and Treatment Strategies. Int. J. Mol. Sci..

[10] Bouras E., Tsilidis K.K., Triggi M., Siargkas A., Chourdakis M., Haidich A.B. (2022). Diet and Risk of Gastric Cancer: An Umbrella Review. Nutrients.

[11] Castelló A., Fernández de Larrea N., Martín V., Dávila-Batista V., Boldo E., Guevara M., Moreno V., Castaño-Vinyals G., Gómez-Acebo I., Fernández-Tardón G. (2018). High adherence to the Western, Prudent, and Mediterranean dietary patterns and risk of gastric adenocarcinoma: MCC-Spain study. Gastric Cancer.

[12] Yin J., Qu J., Liang X., Wang M., Yin J., Qu J., Liang X., Wang M. (2023). Prognostic significance of controlling nutritional status score for patients with gastric cancer: A systematic review and meta-analysis. Exp. Ther. Med..

[13] Tayyem R., Al-Awwad N., Allehdan S., Ajeen R., Al-Jaberi T., Rayyan Y., Bawadi H., Hushki A. (2022). Mediterranean Dietary Pattern is Associated with Lower Odds of Gastric Cancer: A Case-Control Study. Cancer Manag. Res..

[14] Yusefi A.R., Lankarani K.Β., Bastani P., Radinmanesh M., Kavosi Z. (2018). Risk Factors for Gastric Cancer: A Systematic Review. Asian Pac. J. Cancer Prev..

[15] Rosania R., Chiapponi C., Malfertheiner P., Venerito M. (2016). Nutrition in Patients with Gastric Cancer: An Update. Gastrointest. Tumors.

[16] Tapsell L.C., Neale E.P., Satija A., Hu F.B. (2016). Foods, Nutrients, and Dietary Patterns: Interconnections and Implications for Dietary Guidelines. Adv. Nutr..

[17] Wu X., Zhang Q., Guo H., Wang N., Fan X., Zhang B., Zhang W., Wang W., Fang Z., Wu J. (2023). Dietary patterns and risk for gastric cancer: A case-control study in residents of the Huaihe River Basin, China. Front. Nutr..

[18] Rinninella E., Mele M.C., Cintoni M., Raoul P., Ianiro G., Salerno L., Pozzo C., Bria E., Muscaritoli M., Molfino A. (2020). The Facts about Food after Cancer Diagnosis: A Systematic Review of Prospective Cohort Studies. Nutrients.

[19] Maddineni G., Xie J.J., Brahmbhatt B., Mutha P. (2022). Diet and carcinogenesis of gastric cancer. Curr. Opin. Gastroenterol..

[20] Schwingshackl L., Hoffmann G. (2015). Adherence to Mediterranean diet and risk of cancer: An updated systematic review and meta-analysis of observational studies. Cancer Med..

[21] Amiry F., Mousavi S.M., Barekzai A.M., Esmaillzadeh A. (2022). Adherence to the Mediterranean Diet in Relation to Gastric Cancer in Afghanistan. Front. Nutr..

[22] Schwingshackl L., Hoffmann G. (2014). Adherence to Mediterranean diet and risk of cancer: A systematic review and meta-analysis of observational studies. Int. J. Cancer.

[23] Barile J.P., Horner-Johnson W., Krahn G., Zack M., Miranda D., De Michele K., Ford D., Thompson W.W. (2016). Measurement characteristics for two health-related quality of life measures in older adults: The SF-36 and the CDC Healthy Days items. Disabil. Health J..

[24] Craig C.L., Marshall A.L., Sjöström M., Bauman A.E., Booth M.L., Ainsworth B.E., Pratt M., Ekelund U., Yngve A., Sallis J.F. (2003). International physical activity questionnaire: 12-country reliability and validity. Med. Sci. Sports Exerc..

[25] Salahuddin M., Maru T.T., Kumalo A., Pandi-Perumal S.R., Bahammam A.S., Manzar M.D. (2017). Validation of the Pittsburgh sleep quality index in community dwelling Ethiopian adults. Health Qual. Life Outcomes.

[26] Wang Y.-P., Gorenstein C. (2013). Psychometric properties of the Beck Depression Inventory-II: A comprehensive review. Rev. Bras. Psiquiatr..

[27] Tluczek A., Henriques J.B., Brown R.L. (2009). Support for the reliability and validity of a six-item state anxiety scale derived from the State-Trait Anxiety Inventory. J. Nurs. Meas..

[28] Panagiotakos D.B., Pitsavos C., Arvaniti F., Stefanadis C. (2007). Adherence to the Mediterranean food pattern predicts the prevalence of hypertension, hypercholesterolemia, diabetes and obesity, among healthy adults; the accuracy of the MedDietScore. Prev. Med..

[29] Mantzorou M., Tolia M., Poultsidi A., Vasios G.K., Papandreou D., Theocharis S., Kavantzas N., Troumbis A.Y., Giaginis C. (2022). Adherence to Mediterranean Diet and Nutritional Status in Women with Breast Cancer: What Is Their Impact on Disease Progression and Recurrence-Free Patients’ Survival?. Curr. Oncol..

[30] Mizukami T., Piao Y. (2021). Role of nutritional care and general guidance for patients with advanced or metastatic gastric cancer. Future Oncol..

[31] Xu R., Chen X.D., Ding Z. (2022). Perioperative nutrition management for gastric cancer. Nutrition.

[32] Praud D., Bertuccio P., Bosetti C., Turati F., Ferraroni M., La Vecchia C. (2014). Adherence to the Mediterranean diet and gastric cancer risk in Italy. Int. J. Cancer.

[33] Sharma R.N., Sageena G. (2022). Dietary factors associated with gastric cancer—A review. Transl. Med. Commun..

[34] Van den Brandt P.A. (2022). The impact of a healthy lifestyle on the risk of esophageal and gastric cancer subtypes. Eur. J. Epidemiol..

[35] Qiu M., Zhou Y.X., Jin Y., Wang Z.X., Wei X.L., Han H.Y., Ye W.F., Zhou Z.W., Zhang D.S., Wang F.H. (2015). Nutrition support can bring survival benefit to high nutrition risk gastric cancer patients who received chemotherapy. Support Care Cancer.

[36] Dinu M., Pagliai G., Casini A., Sofi F. (2018). Mediterranean diet and multiple health outcomes: An umbrella review of me-ta-analyses of observational studies and randomised trials. Eur. J. Clin. Nutr..

[37] Kyriacou A., Evans J.M., Economides N., Kyriacou A. (2015). Adherence to the Mediterranean diet by the Greek and Cypriot population: A systematic review. Eur. J. Public Health.

[38] Castro-Espin C., Agudo A. (2022). The Role of Diet in Prognosis among Cancer Survivors: A Systematic Review and Meta-Analysis of Dietary Patterns and Diet Interventions. Nutrients.

[39] Steck S.E., Murphy E.A. (2020). Dietary patterns and cancer risk. Nat. Rev. Cancer.

[40] Shin J., Shin D.W., Lee J., Hwang J., Lee J.E., Cho B., Song Y.M. (2022). Exploring socio-demographic, physical, psychological, and quality of life-related factors related with fear of cancer recurrence in stomach cancer survivors: A cross-sectional study. BMC Cancer.

[41] Vajdi M., Farhangi M.A. (2020). A systematic review of the association between dietary patterns and health-related quality of life. Health Qual. Life Outcomes.

[42] Mantzorou M., Koutelidakis A., Theocharis S., Giaginis C. (2017). Clinical Value of Nutritional Status in Cancer: What is its Impact and how it Affects Disease Progression and Prognosis?. Nutr. Cancer.

[43] Sachlova M., Majek O., Tucek S. (2014). Prognostic value of scores based on malnutrition or systemic inflammatory response in patients with metastatic or recurrent gastric cancer. Nutr. Cancer.

[44] Eo W.K., Chang H.J., Suh J., Ahn J., Shin J., Hur J.Y., Kim G.Y., Lee S., Park S., Lee S. (2015). The Prognostic Nutritional Index Predicts Survival and Identifies Aggressiveness of Gastric Cancer. Nutr. Cancer.

[45] Pano O., Gamba M., Bullón-Vela V., Aguilera-Buenosvinos I., Roa-Díaz Z.M., Minder B., Kopp-Heim D., Laine J.E., Martínez-González M.Á., Martinez A. (2022). Eating behaviors and health-related quality of life: A scoping review. Maturitas.

[46] Grosso G., Bella F., Godos J., Sciacca S., Del Rio D., Ray S., Galvano F., Giovannucci E.L. (2017). Possible role of diet in cancer: Systematic review and multiple meta-analyses of dietary patterns, lifestyle factors, and cancer risk. Nutr. Rev..

[47] Behbahani H.B., Borazjani F., Sheikhi L., Amiri R., Angali K.A., Nejad S.B., Samadani M. (2022). The Association between Diet Quality Scores with Sleep Quality among Employees: A Cross-Sectional Study. Ethiop. J. Health Sci..

[48] Scoditti E., Tumolo M.R., Garbarino S. (2022). Mediterranean Diet on Sleep: A Health Alliance. Nutrients.

[49] Bamia C. (2018). Dietary patterns in association to cancer incidence and survival: Concept, current evidence, and suggestions for future research. Eur. J. Clin. Nutr..

[50] Kwon S., Kim J., Kim T., Jeong W., Park E.C. (2022). Association between gastric cancer and the risk of depression among South Korean adults. BMC Psychiatry.

[51] Yu H., Wang Y., Ge X., Wu X., Mao X. (2012). Depression and survival in Chinese patients with gastric cancer: A prospective study. Asian Pac. J. Cancer Prev..

[52] Pan C., Wu J., Zheng S., Sun H., Fang Y., Huang Z., Shi M., Liang L., Bin J., Liao Y. (2021). Depression accelerates gastric cancer invasion and metastasis by inducing a neuroendocrine phenotype via the catecholamine/β2 -AR/MACC1 axis. Cancer Commun..

[53] Han L. (2020). Prevalence, risk factors and prognostic role of anxiety and depression in surgical gastric cancer patients. Transl. Cancer Res..

[54] Liu Y., Chen J., Pan Y., Cai Y., Ge C., Chu H., Xia C., Song Y., Chen Y., Wu B. (2021). The effects of video based nursing education on perioperative anxiety and depression in patients with gastric cancer. Psychol. Health Med..

[55] Rui A., Xu Q., Yang X. (2021). Effect of multidisciplinary cooperative continuous nursing on the depression, anxiety and quality of life in gastric cancer patients. Am. J. Transl. Res..

[56] Wu X., Zhang W. (2023). Reminiscence therapy-based care program alleviates anxiety and depression, as well as improves the quality of life in recurrent gastric cancer patients. Front. Psychol..

[57] Ni H., Sun Y., Meng Y., Zhang J., Yang Y. (2022). Effects of Psychiatric Issues and Early Enteral Nutrition Therapy on Anxiety and Quality-of-Life of Patients with Gastric Cancer. Am. J. Health Behav..

[58] Liu P., Wang Z. (2022). Postoperative anxiety and depression in surgical gastric cancer patients: Their longitudinal change, risk factors, and correlation with survival. Medicine.

[59] Bryniarski P., Bryniarska M., Jezioro M., Andrysiak D., Filipczak-Bryniarska I. (2022). Factors connected with anxiety and other neuropsychiatric symptoms in advanced gastric cancer. Acta Neuropsychiatr..

[60] Zhang L. (2021). Anxiety and depression in recurrent gastric cancer: Their prevalence and independent risk factors analyses. Medicine.

